# Nutrient State-Dependent Ascarosides and Nematode Immune Response Limit the Predation of *Arthrobotrys oligospora*

**DOI:** 10.3390/microorganisms13122816

**Published:** 2025-12-10

**Authors:** Jia-Hong Duan, Zhong-Kan He, Xin-Qian Gong, Qiu Zhao, Xin-Yue Tang, Cheng-Gang Zou, Yi-Cheng Ma

**Affiliations:** 1 State Key Laboratory for Conservation and Utilization of Bio-Resources in Yunnan and Key Laboratory of Industrial Microbial Fermentation Engineering of Yunnan Province, School of Life Sciences, Yunnan University, Kunming 650091, China; jia_9608@163.com (J.-H.D.);; 2 Southwest United Graduate School, Kunming 650092, China

**Keywords:** nematode-trapping fungi, nematodes, *Arthrobotrys oligospora*, ascaroside, innate immunity

## Abstract

Nematode-trapping fungi act as predators of nematodes in soil ecosystems, forming a typical predator–prey relationship. However, this interaction is frequently influenced by environmental factors such as nutrient state. In this study, we demonstrate that starved nematodes had better chances of escaping *A. oligospora* predation by inhibiting *A. oligospora* trap formation. Starved nematodes showed downregulated acyl-CoA oxidase genes (*acox-1.2/1.3/1.4*) and reduced ascaroside pheromone production (ascr#1/#3/#5/#9), thus diminishing *A. oligospora* trap induction. In soils with uneven nutrient content, nutrient deficiencies can activate this mechanism locally, thereby reducing predation. When avoidance fails, nematodes rely on canonical innate immune pathways (FSHR-1, ATFS-1, and PMK-1) to improve survival during capture. In response to this predation, nematodes have evolved multiple strategies to defend against these pressures, closely linked to their nutritional status. Together, these findings link local nutrient availability to both fungal predation efficiency and the robustness of nematode defenses in soil ecosystems.

## 1. Introduction

The interactions between nematodes and nematode-trapping fungi (NTF) are characterized by chemical cues that enable reciprocal sensing and response [1,2,3]. *Caenorhabditis elegans*, a free-living bacterivorous nematode, naturally inhabits microbe-rich and spatially heterogeneous environments, including compost, decaying vegetation, and rhizosphere-associated soils [4,5], where encounters with fungal predators are frequent. *Arthrobotrys oligospora* is a globally distributed NTF that is widely present in agricultural soils, forest floors, grasslands, and organic-rich substrates, and is known to co-occur with diverse free-living nematodes in these habitats [6]. In response to prey-derived ascaroside pheromones, *A. oligospora* forms adhesive traps and modulates its predatory strategies to improve capture efficiency [7]. Besides traps, NTFs use additional predation strategies such as constricting rings, spore-based adhesion in endoparasites, and post-capture effectors (lectins/adhesins, penetration pegs, and lytic enzymes) to immobilize and digest prey [8]. However, the effect of nematodes’ nutritional status on this predation remains to be elucidated.

Ascarosides, hormones produced by *C. elegans*, consist of a dideoxy sugar core with side chains and are synthesized by peroxisomal acyl-coenzyme A oxidase (ACOX) initially. Their length and oxidation state are in turn determined by peroxisomal β-oxidation [9,10,11] to mediate prey-to-prey and prey-to-predator communication [12]. The genome of *C. elegans* encodes seven ACOX genes, five of which (*acox-1.1*, *-1.2*, *-1.3*, *-1.4*, and *acox-3*) are dedicated to isoform-specific substrate preferences to contribute to ascaroside variation and production [13]. ACOX-1.1 and ACOX-3 tend to synthesize medium-to long-chain ascarosides, whereas ACOX-1.2, -1.3, and -1.4 favor shorter chains [14,15,16]. Because cellular lipid catabolism [17] and peroxisomal function shift [18] under nutrient scarcity, this causes the ascaroside profile to undergo changes, which may consequently weaken the chemical cues that activate fungal predation. In natural soils, resources are not evenly distributed but appear as temporary micro-hotspots [19,20], creating patchy patterns of carbon and nutrients [21]. Within these patches, nematode–microbiome interactions depend on changes in resource availability, suggesting that ascaroside production may differ at the microscale. However, the exact ACOX contributors and the specific ascaroside species responsible for *A. oligospora* trapping still remain to be identified.

*C. elegans* possesses conserved innate immune pathways that protect it from microbes, including physical barriers and generated fungicidal materials (reactive oxygen species and antimicrobial peptides) [6,7,22,,23,24,25]. Collectively, the intestinal G protein-coupled receptors (GPCR) FSHR-1 triggers and participates in defensive activity [26,27], the p38 mitogen-activated protein kinases (p38 MAPK) PMK-1 maintains epithelial integrity [28], and the mitochondrial unfolded protein response (UPR^mt^) driven by the transcriptional factor ATFS-1 sustains organellar proteostasis [29]. Although those pathways have been extensively studied in the context of bacterial infection, their roles in resistance to fungal predation remain unclear.

Here, we address the relationship between the nutritional status of nematodes and the predation efficiency of *A. oligospora* and explore which innate immune pathways nematodes rely on to resist predation. We demonstrated that starvation lowered the expression of ACOX genes (*acox-1.2*, *-1.3*, and *-1.4*) and reduced levels of ascaroside (ascr#1, #3, #5, and #9)-induced trap formation. In conditions of abundant nutrient availability, the ability of worms to resist *A. oligospora* predation was found to be contingent upon their canonical innate immune signaling pathways, including FSHR-1, ATFS-1, and PMK-1. This reveals the dual-strategy of nematodes in their natural habitat: when food availability fluctuates between scarcity and abundance, they must continuously switch between ascaroside and innate immune signaling in response to fungal predation.

## 2. Materials and Methods

### 2.1. Strains

The wild-type *Caenorhabditis elegans* strain N2, the mutant strains *daf-16(mu86)*, *hlh-30(tm1978)*, *pmk-1(km25)*, *fshr-1(ok778)*, and *atfs-1(gk3094),* and the transgenic reporter strains (*acox-1.2p::gfp*, *T24B8.5p::gfp*, and *hsp-6p::gfp*) were kindly provided by the Caenorhabditis Genetics Center (CGC; http://www.cbs.umn.edu/CGC, accessed on 15 October 2025), funded by NIH Office of Research Infrastructure Programs (P40 OD010440). All strains were maintained on nematode growth medium (NGM) plates seeded with *Escherichia coli* OP50 at 20 °C under standard conditions. Unless otherwise specified, synchronized young adult worms were used in all experiments. Worms were routinely propagated on 6 cm NGM plates and transferred to fresh OP50-seeded plates every 2–3 days to avoid starvation and overcrowding.

The *Arthrobotrys oligospora* strain used (ATCC 24927) was obtained from the American Type Culture Collection (ATCC). It was cultured on potato dextrose agar (PDA) at 28 °C and subcultured every 7–10 days to maintain vigorous growth and trap-forming ability. For long-term storage, the fungus was grown on PDA plates until abundant sporulation was observed, and spores were gently harvested with sterile water to prepare spore suspensions. The suspensions were aliquoted and stored at −80 °C. For each set of experiments, fresh working plates and spore suspensions were prepared from these stocked cultures to ensure consistency between replicates.

### 2.2. Worm Starvation Treatment

Two types of plates were prepared: nutrient-rich NGM plates seeded with OP50 and nutrient-poor water agar (WA) plates, where fresh *A. oligospora* (1 × 10^5^ spores) were spread and incubated at 28 °C for 3 days before adding worms [30,31,32]. OP50 was prepared according to standard *C. elegans* maintenance protocols, with bacterial density controlled by OD_600_ measurements (OD_600_ ≈ 1.0) [33,34]. For NGM plates, 100–150 μL of OP50 culture was spread evenly on the agar surface and incubated overnight at 20 °C to form uniform lawns. For WA plates, *A. oligospora* spores were obtained from PDA cultures by gently flooding plates with sterile water and filtering through sterile gauze to remove mycelial fragments. The number of spores was adjusted to 1 × 10^5^ per plate in a small volume, which was evenly spread on the WA surface. These plates were incubated at 28 °C for 3 days to allow mycelial growth and the establishment of a pre-predatory network before worms were added. Synchronized young adult worms were washed three times with M9 buffer to remove residual bacteria and then transferred to unseeded NGM plates for 24 h at 20 °C to induce starvation (STR), while control worms were kept on OP50-seeded plates (CTL) with the same density of worms. This treatment generated two groups of worms with different nutritional states but similar handling procedures.

### 2.3. Predation Assay

For the predation assay, 100 synchronized young adult worms were placed on A. oligospora plates and incubated at 20 °C. For each condition, at least three plates were used in each independent experiment. Every 12 h, the number of fungal traps and the percentage of worms captured were counted under a dissecting microscope. Trap density was quantified by counting adhesive traps in several non-overlapping microscopic (Olympus BX51, Olympus Corporation, Tokyo, Japan) fields of fixed area and converting to traps/cm^2^ based on the calibrated field size. Worms were scored as captured when any part of the body was firmly attached to a fungal trap and did not move after gentle prodding with a platinum wire. The assay was carried out using three independent experiments, each starting from independently synchronized worm populations and independently prepared fungal plates.

### 2.4. Relative Quantitative Analysis of Ascarosides in Worms

The CTL and STR worms were placed into pre-weighed 15 mL tubes, washed three times with sterile water, snap-frozen, and then lyophilized for 12 h. Dry mass was recorded to ensure consistency across samples (≥10 mg each). For extraction, 700 µL of 50% methanol was added. The samples were then sonicated in a pre-cooled water bath for 20 min at 50% amplitude with a 6-s-on/6-s-off duty cycle, followed by centrifugation for 15 min at 4 °C at 12,000 rpm. Supernatants were filtered through 0.2 µm PTFE membranes into LC vials (approximately 500–600 µL). UHPLC and HRMS (Orbitrap FTMS) were performed to analyze metabolites using ESI+ in full-scan mode (*m*/*z* 70–1000). Extracted-ion chromatograms (EICs) were generated at exact-mass windows corresponding to the targeted ascarosides 219.0858-219.0880 (ascr#5), 247.1170-247.1194 (ascr#9), 273.1325-273.1353 (ascr#7), 275.1481-275.1509 (ascr#1), and 301.1636-301.1666 (ascr#3). Peaks were integrated with fixed retention-time windows. Relative abundances were derived from peak areas and normalized to the dry mass of each worm sample to allow comparison between CTL and STR groups. The assay was carried out through three independent experiments.

### 2.5. RNA Interference

RNAi bacterial strains containing targeting genes (*acox-1.1*, *acox-1.2*, *acox-1.3*, *acox-1.4*, *acox-3*, *egl-30*, *dbl-1*, *skn-1*, *xbp-1*) were obtained from the Ahringer RNAi library [35]. *E. coli* was cultured in LB containing 100 μg/mL ampicillin at 37 °C for 16 h, and then plated on NGM plates with the same antibiotic and 5 mM IPTG. RNAi bacteria were grown at 25 °C for 16 h. Synchronized L1 larvae were transferred onto plates and kept at 20 °C until they reached young adulthood. Target gene knockdown was achieved by feeding synchronized *C. elegans* larvae with *E. coli* HT115 (DE3) carrying dsRNA vectors (pL4440). Controls received *E. coli* with the empty pL4440 vector.

### 2.6. Quantitative Real-Time PCR (qRT-PCR)

Total RNA was isolated from 100 μL worm suspension using TRIzol reagent (Invitrogen, Waltham, MA, USA) according to the manufacturer’s instructions. cDNA synthesis was performed using the PrimeScript RT reagent kit (Takara, Kusatsu, Japan). qRT-PCR was conducted on a real-time PCR system with SYBR Premix Ex Taq II (Takara). Gene expression levels were normalized to the internal control gene *act-1*, and relative expression was calculated using the 2^−ΔΔCt^ method. Data are presented as the mean fold change ± SD from three independent experiments. The primer sequences used are provided in Table S1.

### 2.7. Survival Assays

For immune pathway screening, about 50 synchronized worms (wild-type, mutants, or RNAi-treated) were transferred to *A. oligospora* WA plates and observed every 12 h. Worms were considered dead when no pharyngeal pumping or movement was observed after gentle prodding. Detailed survival data and statistical analysis are provided in Table S2. Each assay was repeated in three independent biological replicates, with each replicate using 50 nematodes.

### 2.8. Fluorescence Microscopic Analysis

For fluorescence imaging, worms expressing *hsp-6p::gfp*, *acox-1.2p::gfp*, and *T24B8.5p::gfp* were collected in M9 buffer and transferred onto microscope slides. The slides were imaged using a Zeiss Axioskop 2 Plus fluorescence microscope (ZEISS, Oberkochen, Germany). The GFP intensities were analyzed with ImageJ (version 1.47f). All experiments were performed in three independent replicates, with each replicate using 30 nematodes. Identical exposure settings were used within each experiment, and background fluorescence was subtracted before quantification.

### 2.9. Statistical Analysis

Differences in survival rates were analyzed using the log-rank test. Differences in gene expression, trap density, captured fraction, and fluorescence intensity were assessed by performing two-way ANOVA followed by Šidák’s correction for multiple comparisons or Student’s *t*-test. *p*-values of <0.05 were considered statistically significant. Data were analyzed using GraphPad Prism 8 (GraphPad Software Inc., La Jolla, CA, USA).

## 3. Results

### 3.1. Starvation-Induced Ascaroside Reduction in Nematodes Impairs A. oligospora Predation

**Figure 1:**
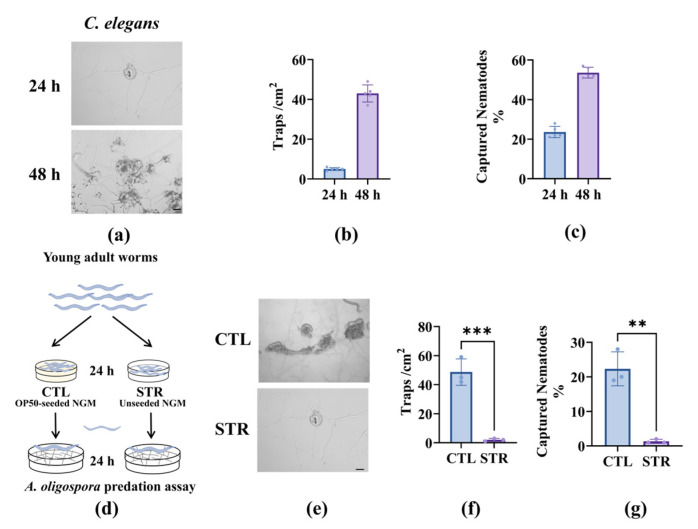
Nematode starvation reduced the predatory capacity of *A. oligospora*: (**a**) Representative images of *C. elegans* at 24 h–48 h on *A. oligospora* plates. Scale bar: 100 μm. (**b**,**c**) Quantification of adhesive trap density (traps/cm^2^) (**e**) and captured fraction (%) (**f**). These results (**b**,**c**) are from three independent experiments. (**d**) Schematic of the starvation (STR) treatment paradigm compared to the fed control (CTL) under the same density of worms, n = 50. Synchronized young adult *C. elegans* were maintained for 24 h on OP50-seeded NGM or on unseeded NGM, then transferred at equal density to *A. oligospora* predation plates. (**e**) Representative images showing fewer adhesive traps surrounding starved worms than controls. Scale bars, 100 μm. (**f**,**g**) Quantification under CTL versus STR, trap density (**f**), and captured fraction (**g**). These results are means ± SD of three independent experiments. ** *p* < 0.01, *** *p* < 0.001. *p*-values (**f**,**g**) were calculated using Student’s *t*-test.

When *C. elegans* nematodes were placed under nutrient-replete conditions, *A. oligospora* trap formation and capture notably increased over the 24–48 h time period (Figure 1a–c). Natural environments are characterized by an uneven distribution of food resources, often fluctuating between scarcity and abundance; therefore, the experiments were designed to evaluate the nematode-killing behavior of *A. oligospora* in nutrient-scarce conditions (Figure 1d). The worms that were subjected to starvation for 24 h prior were surrounded by fewer *A. oligospora* adhesive structures (Figure 1e), with quantitative analyses revealing significant decreases in the number of traps and worms captured compared with well-fed worms (Figure 1f,g). These results suggest that nutrient deprivation in worms compromised the killing behavior and trap formation of *A. oligospora*, resulting in increased nematode survival.

**Figure 2:**
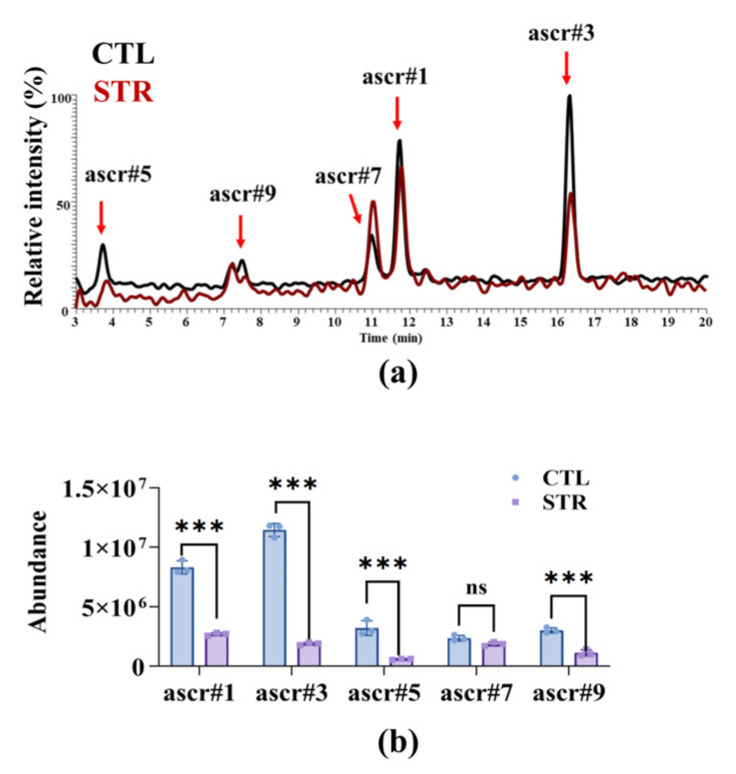
Starvation reduces trap-inducing ascarosides in *C. elegans*: (**a**) Overlaid LC–MS extracted-ion chromatograms (EICs) of conditioned media from feeding controls (CTL, black) and starved worms (STR, red). Arrows mark ascr#5, ascr#9, ascr#7, ascr#1, and ascr#3. (**b**) Quantification of abundance for individual ascarosides. STR significantly decreased ascr#1, ascr#3, ascr#5, and ascr#9 relative to CTL. These results are means ± SD of three independent experiments. ns, no significance, *** *p* < 0.001. *p*-values were calculated using two-way ANOVA with Šidák’s correction for multiple comparisons.

Ascaroside has been identified as the most significant chemical signal for triggering the formation of traps by nematode-trapping fungi [11,36]. To investigate how starvation affects worms and thereby reduces fungal predation capacity, we analyzed ascaroside levels (Figure 2a), identifying significantly lower peak intensities for ascr#1, ascr#3, ascr#5, and ascr#9 in starved worms compared to well-fed worms (Figure 2b). However, the level of ascr#7 remained unchanged in worms after starvation (Figure 2b). This result suggests that under nutrient-poor conditions, nematodes are unable to synthesize ascaroside to an adequate extent.

### 3.2. Starvation Downregulation of the ACOX Genes in Nematodes Reduces the Predation Efficiency of A. oligospora

**Figure 3:**
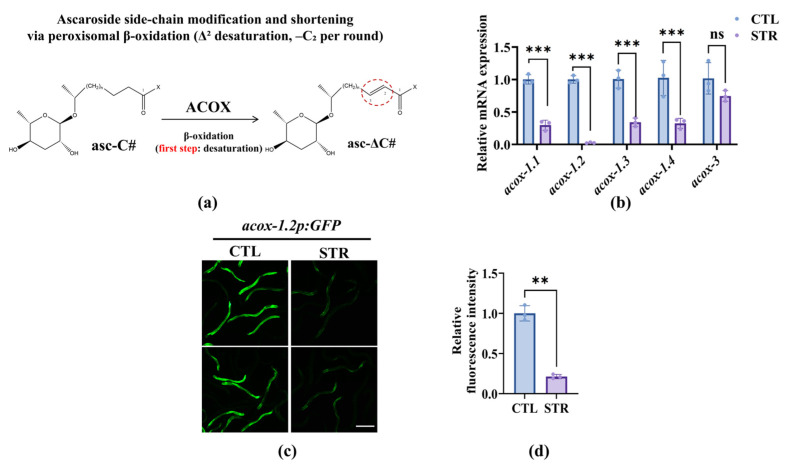
Nutritional stress represses the mRNA Level of the *acox-1* isoforms: (**a**) Schematic of ascaroside side-chain editing/shortening via peroxisomal β-oxidation (first step). ACOX denotes acyl-CoA oxidase in the β-oxidation cycle. (**b**) The mRNA level of *acox* isoform genes. STR reduced *acox-1.1*, *acox-1.2*, *acox-1.3*, and *acox-1.4* mRNA, whereas *acox-3* was unchanged. These results are means ± SD of three independent experiments. ns, no significance, *** *p* < 0.001. *p*-values were calculated using two-way ANOVA with Šidák’s correction for multiple comparisons. (**c**) Representative fluorescence images of the transcriptional reporter *acox-1.2p::GFP* in CTL and STR animals. Scale bar, 100 µm. (**d**) Quantified reporter intensity shows decreased *acox-1.2* promoter activity under STR. These results are means ± SD of three independent experiments (n = 30 per group). ** *p* < 0.01. *p*-values were calculated using Student’s *t*-test.

Peroxisomal β-oxidation is known to modify ascaroside side chains via a set of ACOX genes (Figure 3a), which display chain-length-specific activity on ascaroside precursors [13,15,37]; therefore, we determined which of the ACOX genes responded to starvation. qRT-PCR showed that the mRNA levels of *acox-1.1*, *acox-1.2*, *acox-1.3*, and *acox-1.4* were reduced in starved worms; however, *acox-3* remained steady (Figure 3b). Consistently, the expression of *acox-1.2p::gfp* displayed a lower fluorescence intensity in starved worms than in well-fed worms (Figure 3c,d). These results suggest that *acox-1.1*, *-1.2*, *-1.3*, and *-1.4* are potential effectors for reducing trap-inducing ascaroside production in nematodes.

**Figure 4:**
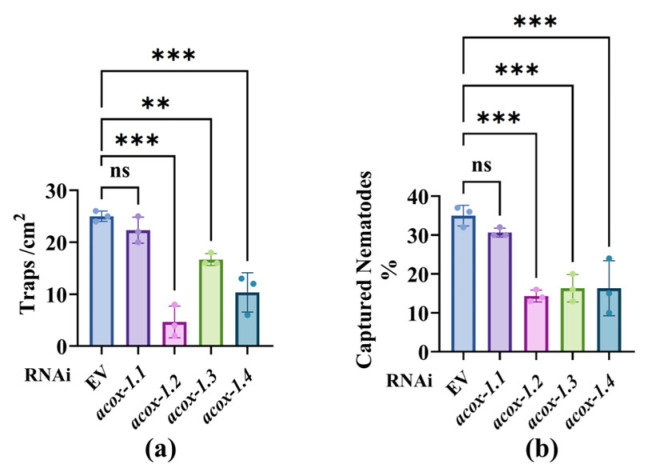
ACOX-1 regulated the trapping and capture behavior of *A. oligospora* under feeding conditions: (**a**) Trap density on *A. oligospora* plates for EV and isoform genes RNAi (*acox-1.1*, *acox-1.2*, *acox-1.3*, *acox-1.4*). (**b**) Quantification of captured fraction (%). These results are means ± SD of three independent experiments. ns, no significance, ** *p* < 0.01, *** *p* < 0.001. *p*-values (**a**,**b**) were calculated using one-way ANOVA with Dunnett’s multiple-comparison correction test.

Knockdown of *acox-1.2*, *acox-1.3*, and *acox-1.4* by RNAi in well-fed worms reduced the number of traps created and the number of worms captured by *A. oligospora* (Figure 4a,b). However, knockdown of *acox-1.1* had no detectable effects in this regard (Figure 4a,b). Furthermore, knockdown of *acox-1.2* in starved worms failed to cause a further decrease in both the number of traps and the number of worms captured (Figure S1). Consequently, we identified *acox-1.2*, *acox-1.3*, and *acox-1.4* as effectors that responded to starvation and modulated the predation efficiency of *A. oligospora*.

### 3.3. Nematodes Rely on Canonical Innate Immune Pathways to Improve Their Survival After the Capture by A. oligospora

**Figure 5:**
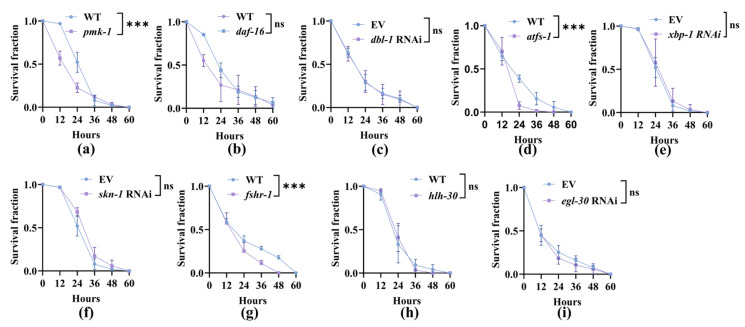
The worms depend on PMK-1, ATFS-1, and FSHR-1 for survival during *A. oligospora* Predation: (**a**–**i**) Kaplan–Meier survival fractions of *C. elegans* exposed to *A. oligospora*. During *A. oligospora* predation, survival required *pmk-1* (**a**), *atfs-1* (**d**), and *fshr-1* (**g**), whereas *daf-16*, *dbl-1* (RNAi), *xbp-1* (RNAi), *skn-1* (RNAi), *hlh-30*, and *egl-30* (RNAi) were dispensable (**b**,**c**,**e**,**f**,**h**,**i**). Kaplan–Meier curves with WT or EV controls as indicated. Shown is a representative experiment of three independent repeats (n = 100 per group). ns, no significance, *** *p* < 0.001. *p*-values (**a**–**i**) were calculated by two-sided log-rank (Mantel–Cox) tests.

Previous research has shown that fungi activate conserved innate responses in worms, including p38 MAPK [38,39], insulin/IGF-1 signaling (DAF-16/FOXO), reactive oxygen species [22], and antimicrobial peptides production [40]. We aimed to determine which canonical innate immunity pathways were involved in resistance to *A. oligospora*. The survival rate of the mutant worm strains *daf-16 (mu86)*, *hlh-30 (tm1978)*, *pmk-1 (km25)*, *fshr-1 (ok778)*, and *atfs-1 (gk3094)*, and those subjected to RNAi (*dbl-1*, *xbp-1*, *skn-1*, and *egl-30*), was measured after exposure to *A. oligospora* (Figure 5 and Table S2). We found that worm survival was dependent on p38 MAPK, as *pmk-1* mutants showed a reduced survival rate (Figure 5a). In contrast, neither DAF-16/FOXO nor TGF-β/DBL-1 altered the survival rate of worms after exposure to *A. oligospora* (Figure 5b,c). The mitochondrial unfolded protein response (UPR^mt^) was found to be necessary for survival on *A. oligospora*, as the loss of *atfs-1* reduced the worm survival rate (Figure 5d), whereas the endoplasmic reticulum unfolded protein response (UPR-ER) XBP-1 and regulator of the cellular stress response SKN-1 did not contribute to survival (Figure 5e,f). The ortholog of the mammalian glycopeptide hormone receptor, FSHR-1, was also required for survival (Figure 5g), while the conserved basic helix–loop–helix transcription factor, HLH-30/transcription factor EB (TFEB), and the vertebrate heterotrimeric G protein alpha subunit, EGL-30/Gq, did not alter survival (Figure 5h,i). Taken together, our data indicated that PMK-1, ATFS-1, and FSHR-1 were the canonical innate immunity pathways that enabled the worms to resist *A. oligospora*.

**Figure 6:**
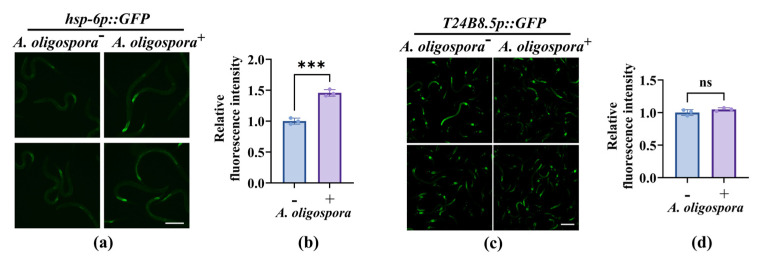
The expression of UPR^mt^ marker increased upon exposure to *A. oligospora*: (**a**) *hsp-6p::GFP* fluorescence in *C. elegans* exposed to *A. oligospora* (+) versus unexposed (−). Scale bar: 100 μm. (**b**) Quantification of reporter fluorescence intensity increased upon exposure. These results are means ± SD of three independent experiments. *** *p* < 0.001. (**c**) Representative images of *T24B8.5p::GFP*. Scale bar: 200 μm. (**d**) Quantification of fluorescence intensity. These results are means ± SD of three independent experiments (n = 30 per group). ns, no significance. *p*-values (**b**,**d**) were calculated using Student’s *t*-test.

The transgenic worms that carried *hsp-6p::gfp* [41] and *T24B8.5p::gfp* [42,43] were used as markers of UPR^mt^ and p38 MAPK, respectively. We found that the GFP intensity of HSP-6 was significantly increased in worms after exposure to *A. oligospora* (Figure 6a,b). However, the GFP intensity of T24B8.5 was unchanged in worms after exposure to *A. oligospora* (Figure 6c,d), demonstrating that *A. oligospora* activated UPR^mt^ but may not have activated p38 MAPK. The requirement for PMK-1, despite the absence of *T24B8.5* induction by *A. oligospora*, suggests that p38 MAPK functions as a basal defense for worms against *A. oligospora*.

## 4. Discussion

Our findings reveal that nematode nutritional state shapes interactions with nematode-trapping fungi at two levels: by altering ascaroside-based prey cues and by selectively engaging innate immune pathways during fungal attack. Natural soils show considerable variation over space and time with regard to nutrient and carbon levels, creating localized nutrient-rich zones that impact microbial activity and species interactions [19,20]. Under laboratory nutrient-limiting conditions, we found that this dietary state change lowered ascaroside output in nematodes, which in turn reduced the trap induction and predatory activity of *A. oligospora*. Starvation represses *acox-1.2/1.3/1.4*, selectively depleting short-chain ascarosides (ascr#1/#3/#5/#9) rather than uniformly reducing all congeners. During the *A. oligospora* attack, worm survival was contingent on the innate immune pathways of FSHR-1, ATFS-1, and PMK-1. In combination, these diet-sensitive changes in pheromone production and host defense modulate fungal trap formation and nematode survival. The dual modulation of prey cues and immunity is a mechanism that may help account for site-to-site variability in NTF-based biocontrol.

In nematodes, ascarosides are compositionally complex mixtures of congeners that differ in side-chain length and head-group modifications. Shifts in ascaroside composition modulate well-defined nematode behaviors and interspecies communications, including dispersal, aggregation, mating, foraging, and stress adaptation, which are strongly conditioned by diet and developmental stage [15,44,45]. Among these, short-chain molecules, notably ascr#1, ascr#3, ascr#5, and ascr#7, and related congeners, function as strong prey cues that trigger trap morphogenesis in NTFs [13,14,15]. Consistent with this, our results demonstrate that nutrient limitation specifically resulted in the downregulation of *acox-1.2*, *acox-1.3*, and *acox-1.4*, thereby shifting biosynthesis away from short-chain species in worms. This caused a decrease in ascr#1, ascr#3, ascr#5, and ascr#9, and subsequently the reduced trap induction and capture behavior of *A. oligospora*.

In agricultural and natural ecosystems, nematode-trapping fungi have been explored as biological control agents to suppress plant-parasitic nematodes [8,46,47]. However, the efficacy of NTF-based biocontrol is notoriously variable across field sites and seasons, often performing well in some soils but not in others [8]. Our results provide a mechanistic explanation for part of this variability. Because short-chain ascarosides that induce trap formation are selectively reduced under nematode starvation, the nematode populations inhabiting nutrient-poor habitats may produce low levels of ascaroside signals, making them less vulnerable to predation by *A. oligospora*. In contrast, the nematode populations inhabiting nutrient-rich habitats may produce stronger ascaroside signals, thereby enhancing trap formation by *A. oligospora*. Thus, microscale nutrient heterogeneity can create a shifting mosaic of “high-risk” and “low-risk” zones for nematodes, with consequences for both nematode community structure and the realized impact of NTFs as biocontrol agents.

Furthermore, in *C. elegans*, the PMK-1/p38-ATF-7 pathway is required for survival and broad effector induction during *Pseudomonas aeruginosa* 14 (PA14) infection [48]. The GPCR FSHR-1 promotes host defense across Gram-negative and Gram-positive challenges in parallel to p38 [26], while the mitochondrial UPR regulator ATFS-1 mediates pathogen-induced mitochondrial stress to innate immunity and enhances survival under PA14 infection [49]. Furthermore, some stress responses, like HLH-30, are required for protection during *Staphylococcus aureus* infection and mediate responses to bacterial pore-forming toxins [50]. SKN-1 is activated via TIR-1/PMK-1 during PA14 or *Enterococcus faecalis* infection [51]. DAF-16/FOXO improves survival when insulin/IGF-1 signaling is reduced across PA14, *S. aureus*, or *E. faecalis* [52]. In addition, DBL-1/TGF-β signaling modulates antimicrobial peptide expression during *Drechmeria coniospora* infection [53]. Beyond immune and stress response programs, cuticle-trap adhesion is a further biophysical determinant during predation by nematode-trapping fungi. Forward genetic screening identified NHR-66 as a regulator of cuticular collagens, whose loss reduces trapping across multiple *Arthrobotrys* species [3]. These studies together indicate that multiple immune, stress, and cuticular pathways can influence nematode survival under diverse pathogenic and predatory challenges.

Building on these mechanisms, our identification of FSHR-1, ATFS-1, and PMK-1 as key factors for nematode survival during *A. oligospora* predation indicates that host defense is not a generalized innate immunity response. Instead, it is mediated by specific innate immune pathways initially characterized in bacterial infection models. We found that UPR^mt^, but not all other stress response regulators like SKN-1 or XBP-1, are essential for the survival of worms, suggesting that mitochondria are especially crucial during fungal trapping and digestion. This may reflect mitochondria’s dual function as centers for metabolic adaptation to starvation and for integrating immune signals. In addition, the basal activity of PMK-1 also contributes to nematode survival during fungal trapping and digestion. Future research exploring downstream effector genes of these pathways in NTF predation will help clarify how nematodes balance immune responses with other starvation-related adaptations. These results demonstrate that during exposure to *A. oligospora*, worms deploy specific defense response pathways rather than a generalized immune response.

## 5. Conclusions

Our findings demonstrate a two-strategy nematode defense model whereby nutrient limitation downregulates the expression of acyl-CoA oxidase genes to selectively deplete the short-chain ascarosides that activate nematode-trapping fungi, while post-capture survival requires several focused innate immune pathways (PMK-1, ATFS-1, and FSHR-1). These results suggest that microscale nutrient patchiness modulates fungal predation pressure in situ, offering a framework for interpreting variability in field biocontrol performance. This dual-defense strategy has significant theoretical value for research into bioactivated nematicides.

## Data Availability

The original contributions presented in this study are included in the article/Supplementary Materials. Further inquiries can be directed to the corresponding authors.

## Supplementary Materials

The following supporting information can be downloaded at: https://www.mdpi.com/article/10.3390/microorganisms13122816/s1, Figure S1: No significant effect of *acox-1.2* RNAi in *A. oligospora* trap formation in STR; Table S1: Primers of qPCR analysis; Table S2: Survival assay data; Table S3: The fold change of gene level and survival of *C. elegans* impact during *A. oligospora* predation.
