# Supplementary material for: Nutrient State-Dependent Ascarosides and Nematode Immune Response Limit the Predation of Arthrobotrys oligospora
Source: Microorganisms. 2025 Dec 10;13(12):2816. doi: 10.3390/microorganisms13122816 (PMC12735411; doi:10.3390/microorganisms13122816)
Supplement: Supplementary file 1 [file microorganisms-13-02816-s001.zip › Figure S1.pdf]

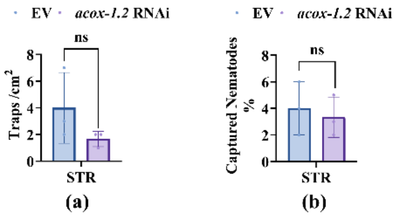

Figure S1. No Significant Effect of *acox-1.2* RNAi in *A. oligospora* Trap Formation in STR. (**a**) Trap density on *A.oligospora* lawns for EV and *acox-1.2* RNAi. (**b**) Quantification of captured fraction (%). These results are means ± SD of three independent experiments. ns, no significant. *P*-values (a and b) were calculated using Student's t-test.
